# Metformin in cardiovascular diabetology: a focused review of its impact on endothelial function

**DOI:** 10.7150/thno.64706

**Published:** 2021-09-09

**Authors:** Yu Ding, Yongwen Zhou, Ping Ling, Xiaojun Feng, Sihui Luo, Xueying Zheng, Peter J. Little, Suowen Xu, Jianping Weng

**Affiliations:** 1 Institute of Endocrine and Metabolic Diseases, The First Affiliated Hospital of USTC, Division of Life Sciences and Medicine, University of Science and Technology of China, Hefei, China.; 2 Department of Pharmacy, The First Affiliated Hospital of USTC, Division of Life Sciences and Medicine, University of Science and Technology of China, Hefei, China.; 3 Division of Life Sciences and Medicine, University of Science and Technology of China, Hefei, China.; 4 Sunshine Coast Health Institute, University of the Sunshine Coast, Birtinya, QLD 4575, Australia.; 5 School of Pharmacy, Pharmacy Australia Centre of Excellence, the University of Queensland, Woolloongabba, Queensland 4102, Australia.

**Keywords:** Metformin, cardiovascular diabetology, endothelial function, diabetes, panvascular disease

## Abstract

As a first-line treatment for diabetes, the insulin-sensitizing biguanide, metformin, regulates glucose levels and positively affects cardiovascular function in patients with diabetes and cardiovascular complications. Endothelial dysfunction (ED) represents the primary pathological change of multiple vascular diseases, because it causes decreased arterial plasticity, increased vascular resistance, reduced tissue perfusion and atherosclerosis. Caused by “biochemical injury”, ED is also an independent predictor of cardiovascular events. Accumulating evidence shows that metformin improves ED through liver kinase B1 (LKB1)/5'-adenosine monophosphat-activated protein kinase (AMPK) and AMPK-independent targets, including nuclear factor-kappa B (NF-κB), phosphatidylinositol 3 kinase-protein kinase B (PI3K-Akt), endothelial nitric oxide synthase (eNOS), sirtuin 1 (SIRT1), forkhead box O1 (FOXO1), krüppel-like factor 4 (KLF4) and krüppel-like factor 2 (KLF2). Evaluating the effects of metformin on endothelial cell functions would facilitate our understanding of the therapeutic potential of metformin in cardiovascular diabetology (including diabetes and its cardiovascular complications). This article reviews the physiological and pathological functions of endothelial cells and the intact endothelium, reviews the latest research of metformin in the treatment of diabetes and related cardiovascular complications, and focuses on the mechanism of action of metformin in regulating endothelial cell functions.

## Diabetes and cardiovascular disease - the dangerous liaison

Patients with diabetes suffer from a higher incidence and rate of mortality of cardiovascular disease (CVD) compared with nondiabetic subjects 1. Diabetes *per se* is also a high-risk factor for CVD 2. According to the disease statistic report from the International Diabetes Federation, the global prevalence of diabetes was 9.3% in 2019 estimated and is projected to reach 10.2% by 2030 3. Numerous studies have shown that there is a positive correlation between glucose levels and the incidence of CVD. It is reported that the incidence of heart failure increases by 15% when glycosylated hemoglobin increases by 1%, and the incidence of myocardial infarction decreases by 16% if anti-hyperglycemic drugs are used intensively 4, 5. With multiple pathophysiological components, including hyperglycemia, insulin resistance (IR), hyperinsulinemia and hyperlipidemia, diabetes changes the vascular responsiveness to various vasoconstrictors and vasodilators and is a vital factor in the occurrence and development of the vascular complications of diabetes 5. Hyperglycemia is the primary determinant factor of thrombosis in the coronary vessels through the modification of microbiota thrombus colonization in hyperglycemic patients with ST-segment elevation myocardial infarction (STEMI) 6, and via the direct modification of atherosclerotic plaque instability and rupture with consequent acute thrombosis (in STEMI) and worse prognosis 7, 8. The overexpression of inflammatory and pro-thrombotic markers is linked to the hyperglycemic levels in the serum, the coronary thrombus 9 and the peri-coronary fat 10. This is evidence of excessive inflammation and pro-thrombotic effects of hyperglycemia in patients with atherosclerotic plaque instability, rupture, acute thrombosis, and STEMI.

Various diabetes-related pathological changes vary with the size and location of blood vessels, leading to decreased arterial plasticity, increased vascular resistance, reduced tissue perfusion, and atherosclerosis 11, 12. In the process of atherosclerotic plaque formation, circulating monocytes first adhere to the lesion area of the vascular wall, then infiltrate into the wall, and differentiate into macrophages. These macrophages take up modified lipids through phagocytosis and scavenger receptor mediated processes, and leading to the formation of foam cells, which represent the hallmark of arterial plaques 13-16. Based on these pathological changes, the development and progression of atherosclerosis plays a critical role in the underlying pathological basis of most patients with stable coronary disease 17.

Ross and colleagues proposed the “endothelial cell injury” model of atherosclerosis (“response to injury”) sort of assuming that atherosclerosis results from physical damage to the endothelium 18. It was subsequently found that biochemical molecules 19, 20, including turbulent blood flow and inflammatory cytokines/chemokines, actually serve as physiochemical injury. We propose the hypothesis that “biochemical injury” is the cause of endothelial dysfunction (ED). ED is related to a series of pathological events in diabetes and its complications 21. In patients with stable coronary disease, with the increase of IR and carbohydrate intolerance, the functional coronary circulation abnormalities of endothelium-dependent vascular movement will gradually worsen 22. Impaired NO-dependent vasodilatation is a predictor of future cardiac events and the development of coronary ED 23. Moreover, coronary ED is as a cause of ischemic heart disease (IHD) linked worse prognosis in diabetic patients with stable coronary disease 24, 25. This is evidenced in stable IHD patients during angiography of coronary vessels and invasive measurements of ED. It is noteworthy that the occurrence of ED and the development of atherosclerosis is very rapid in patients with diabetes in the absence of an effective pharmacological intervention 26. ED is also linked to insulin resistant states including obesity, diabetes, and the metabolic dysfunction 27. Hyperglycemia is the major causal factor in the development of ED in patients with diabetes. Hyperglycemia is also a main risk factor for CVD, particularly IHD alone and in combination with diabetes. Indeed, hyperglycemia is a risk factor for CVD/IHD independently from diabetes.

Diabetes can be classified into several categories, including type 1 diabetes (T1D), type 2 diabetes (T2D), gestational diabetes mellitus, and specific types of diabetes due to other causes. Accounting for 90 - 95% of all diabetes, T2D is generally due to a progressive loss of β-cell insulin secretion frequently on the background of IR 28. The established relationship between T2D and CVD provides a therapeutic framework that anti-diabetic drugs may have beneficial effects on cardiovascular risk factors and help reduce the atherosclerotic process in diabetic patients. In addition to hypoglycemic benefits, whether anti-diabetic drugs can prevent or treat ED and improve atherosclerosis and CVD is an important research direction 29, 30. When choosing pharmacological interventions for patients with the comorbidities of diabetes and CVD, the hypoglycemic effects and potential cardiovascular benefits should be considered simultaneously. These considerations are critical to our understanding of the potential role of antidiabetic medications in reducing the development and progression of atherosclerosis and CVD in patients both with and without diabetes.

Because the treatment of hyperglycemia changes many factors other than circulating glucose levels, clinical and experimental data support the hypothesis that factors other than hyperglycemia are the culprit 31. As the first-line drug for most patients with T2D, metformin is indicated because of its benefits on glycemia management but also affects several other pathways/mechanisms including oxidative stress, inflammation, lipoprotein metabolism and thrombosis 32, 33. For example, metformin provides a protective effect on cardiovascular function by reducing weight gain and serum-free insulin level, according to the report from UK Prospective Diabetes Study (UKPDS) 34. Moreover, various studies have emphasized on the anti-inflammatory and antioxidant role of antidiabetic medications, with multiple mechanisms, which activation of 5'-adenosine monophosphat-activated protein kinase (AMPK) by metformin has had a key role in many of them 35-37. Notably, the pleiotropic effects of antidiabetic medications have been seen for metformin and new classes of antidiabetic drugs and incretins. To date, metformin shows pleiotropic effects to reduce the peri-coronary inflammatory stress 10, including anti-thrombotic and anti-atherosclerotic properties, which are also played by incretins in STEMI 38 and non-STEMI 39 patients. Therefore, this evidences the protective effects played by incretins on the atherosclerotic plaque for patients with acute coronary syndrome, in the condition of acute coronary thrombosis 38 and multivessel disease with and ED 39. For a detailed review of cardiovascular actions and molecular targets of glucagon-like peptide-1 receptor agonists (GLP-1RAs) 36 and sodium glucose cotransporter 2 inhibitors (SGLT2i) 37, please refer to our recent reviews. As mentioned above, ED can be an independent predictor of cardiovascular events and the first step toward coronary arteriosclerosis 40. Therefore, evaluating the effects of metformin on improving ED would deepen our knowledge of the therapeutic potential of metformin. In this review, we aim to provide a comprehensive account of the physiological and pathological functions of endothelial cells and the intact endothelium, and summarize the latest research on metformin in diabetes and associated cardiovascular complications, with particular emphasis on the role of metformin on regulating endothelial cell functions.

## Endothelial cell functions

**Figure 1:**
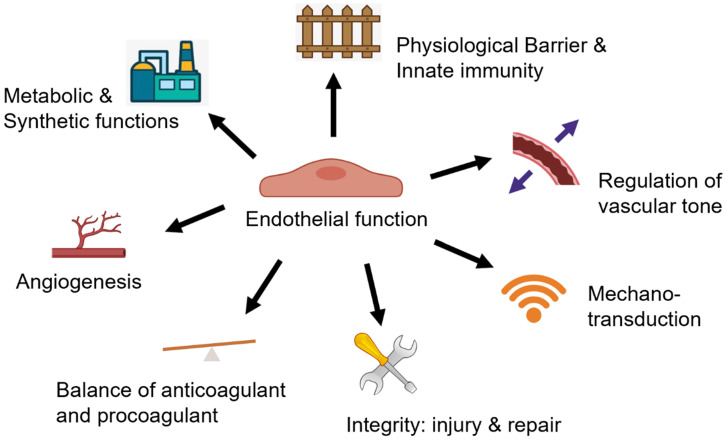
** Multi-faceted biological functions of endothelium.** The healthy endothelium serves diverse biological functions in physiological state, including 1) serving as a physiological barrier and site of innate immunity; 2) regulation of vascular tone; 3) mediating mechanotransduction through mechanical sensors/mechanosensitive complexes; 4) keeping endothelial integrity by an effect on both endothelial injury and the capacity for endothelial repair; 5) keeping the balance of anticoagulant and procoagulant function; 6) regulating angiogenesis; 7) as an essential cell type for the metabolic and synthetic function.

There are three layers of blood vessels, and the innermost layer is the intima composed of a monolayer of endothelial cells and the underlying internal elastic lamella 41. In the physiological state, the healthy endothelium serves diverse biological functions (Figure 1) and creates an adaptive life support system that extends to almost every tissue and organ 42.

Among all the functions of the intact endothelium 43, the first is its role as a physiological semi-permeable barrier and an emerging component of innate immunity. As the barrier and site of innate immunity between blood and tissues, the endothelium actively regulates the exchange and transport of small and large molecules and defends against invading pathogens and infections 44, 45. In addition, as a carbohydrate-rich layer lining the vascular endothelium 46, the glycocalyx acts as a barrier between blood, the endothelium and underlying tissues and plays an essential role in maintaining the barrier integrity and consequently homeostasis and quiescence of the vasculature 47.

A pivotal function of the endothelium is to regulate vascular tone through secreting substances that relax and constrict blood vessels. Nitric oxide (NO), a classical mediator of vasodilation, is produced by endothelial cells and glycocalyx. NO regulates the relaxation of vascular smooth muscle cells (VSMCs) mainly by stimulating soluble guanylyl cyclase to produce cyclic guanosine monophosphate 46, 48. By reacting with the Cys thiol group and reversibly regulating S-nitrosylation, NO can play a role in relaxing blood vessels 49. Endothelial nitric oxide synthase (eNOS) is the leading source of most vascular NO, and is also a critical enzyme to protect endothelium against vascular damage 50, 51. Besides NO, vasodilators include other dissolved gasses such as hydrogen sulfide (H_2_S) and carbon monoxide (CO), which are also secreted by endothelial cells 52-54. The endothelium also produces vasoconstrictor molecules, such as angiotensin II (AngII), endothelin 1 (ET-1), thromboxane A_2_ (TXA_2_), thrombin, and others 55. The dynamic balance between vasoconstrictor and vasodilator mediators is an important foundation for endothelial cells to maintain homeostasis. On the other hand, the autonomic nervous system and various neurotransmitters play an essential role in the regulation of vascular tone 56. The endothelial cells express a variety of receptors that specifically interact with neurotransmitters from the nerve terminal. Among various neurotransmitters, norepinephrine (NE), adenosine triphosphate (ATP) and neuropeptide Y (NPY) relax blood vessels, while acetylcholine (Ach) and calcitonin gene-related peptide (CGRP) act as vasodilators. The endothelial cells are triggered to produce NO and other vasoactive gases when the adrenergic endothelial receptors are activated 57.

The endothelium senses mechanical stress and mediates mechano-transduction through mechanosensors or mechanosensitive complexes 58. Endothelial cells convert mechanical stress into intracellular biochemical signals, such as increased NO, H_2_S, and calcium concentration 59-61. Thus, these responses of vascular mechano-transduction serve to prevent vascular cells from damage induced by mechanical injury 58. Endothelial integrity, another primary function of the endothelium, is affected by the degree of injury and depends on the integrity of the endogenous repair ability of the vessels 62. When an adjacent endothelial cell is damaged, the local mature endothelial cell can replicate to replace the lost and damaged cell. However, if endothelial cells only rely on local replication function, it is far from enough to resist external damage 63. A recently study shows that the protection is driven by a transient activation of extracellular-signal-regulated kinase (ERK) in endothelial cells neighboring extruding cells, which inhibits caspase activation and prevents elimination of endothelial cells in clusters 64. Endothelial progenitor cells (EPCs) in the circulation participate in another repairing mechanism for maintaining the integrity of the endothelium, which has been clearly explained 65. Therefore, mechano-transduction and circulating EPCs may play an essential role in the pathogenesis of vascular disease by affecting endothelial injury and repair.

Maintaining the balance between anticoagulant and procoagulant status of the vessel wall and blood is a critical function of the endothelium. Although endothelial cells have close contact with platelets, there is no harmful reaction in the blood vessels of a healthy organism 66. Plasminogen activator (PA) and plasminogen activator inhibitor (PAI) are synthesized and secreted by endothelial cells, providing anticoagulant and procoagulant regulatory mechanisms, respectively 67. There are several mechanisms involved in the regulation process, such as enhanced plasminogen activation by tissue-PA (t-PA) or urokinase-PA (u-PA), reduced inhibition of plasmin by α2-antiplasmin or of PAs by PAIs, and enhanced conversion of single-chain u-PA to two chain u-PA 68.

Angiogenesis, depending on endothelial cell migration, is another basis function of the endothelium 69. Endothelial cells regulate angiogenesis through producing several factors, including vascular endothelial growth factor (VEGF), NO, matrix metalloproteinases (MMPs), tissue inhibitors of metalloproteinases (TIMPs), and triggers 44, 70. On this basis, endothelial cells have the ability to adjust their number and arrangement to meet local needs.

As a sizeable endocrine organ, endothelial cells have a metabolic (large source of energy form glycolysis) and synthetic function. In addition to vasoactive substances, endothelial cells can also secrete various mediators, thus affecting the whole-body cell function 44. Endothelial cells are also processing factories of important metabolites, such as glucose, fatty acids, amino acids, lipids, ATP, acetyl coenzyme A, and lactate, and endothelial cells regulate the supply of nutrition and oxygen to all tissues of the body 44, 71. The angiocrine and angiogenic function of endothelial cells underlie its importance in regulating tumor metastasis and neovascularization in states of myocardial infarction.

In summary, these physiological functions of the intact endothelium complement each other, and each role is crucial to maintaining the homeostasis of the vasculature.

## Endothelial dysfunction (ED) in diabetes and its complications

### Hyperglycemia and ED

**Figure 2:**
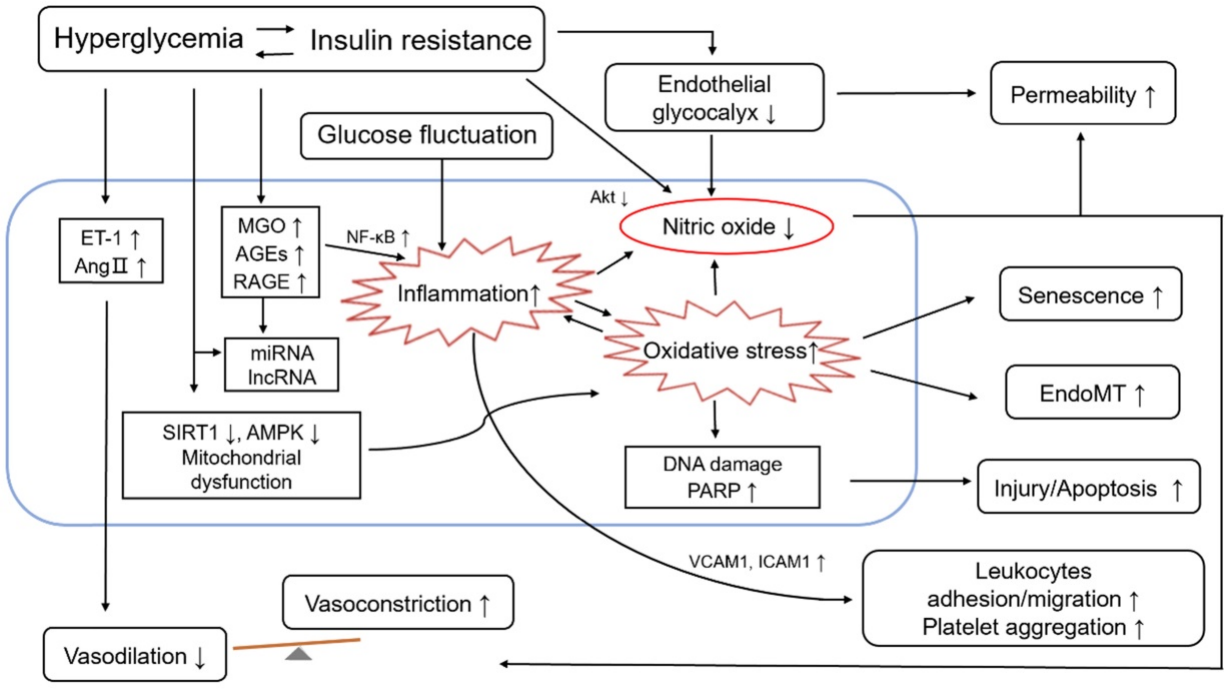
** Role of endothelial dysfunction in diabetes and its cardiovascular complications.** Hyperglycemia is the leading and common characteristic of diabetes and its cardiovascular complications, including insulin resistance and glucose fluctuation. Hyperglycemia drives endothelial dysfunction via different mechanisms, such as 1) Decreased bioavailability of NO and increased secretion of vasoconstrictors, e.g., ET-1 and Ang II; 2) Increased level of MGO/AGE/RAGE; 3) Enhanced inflammatory response, which is also affected by increased glucose fluctuation; 4) Enhanced oxidative stress; 5) Disruption of endothelial glycocalyx; 6) The expression of epigenetic, such as the level some miRNA, lncRNA. The role of endothelial dysfunction is reflected in the following aspects: 1) The vasodilation decreases and the vasoconstriction increases, and then the vascular homeostasis is broken; 2) Leukocytes adhesion and migration, and platelet aggregation increase; 3) The injury/apoptotic process of endothelial cells is increased; 4) The process of EndoMT is promoted; 5) The endothelial senescence is increased; 6) The permeability of endothelial cells increases. Abbreviations: AMPK, 5'-adenosine monophosphate-activated protein kinase; AGEs, advanced glycation end products; Ang II, angiotensin II; EndoMT, endothelial-to-mesenchymal transition; ET-1, endothelin 1; ICAM1, intercellular adhesion molecule 1; LncRNAs, long noncoding RNAs; MGO, methylglyoxal; MiRNAs, microRNAs; NF-κB, nuclear factor-KappaB; PARP, poly ADP-ribose polymerase; Akt, also known as PKB, protein kinase B; RAGE, receptor for AGEs; SIRT1, sirtuin 1; VCAM1, vascular cell adhesion molecule 1.

ED occurs when the production and secretion of vasodilators and vasoconstrictors are out of balance, leading to impaired endothelium-dependent vasodilation. Hyperglycemia, as the defining clinical feature of diabetes mellitus, is known to cause ED through several mechanisms 72, including those involving advanced glycation end products (AGEs), hexosamine, glycocalyx reduction, protein kinase C (PKC), oxidative stress, proliferative dysfunction, ascorbic acid and aldose reductase 73-78. Hyperglycemia induces changes in a variety of cellular signaling and metabolic pathways including altered hemodynamics due to decreased eNOS activity and increased ET-1 synthesis, increased oxidative stress and inflammation, basement membrane thickening caused by transforming growth factor-β (TGF-β) mediated increased synthesis of collagen IV and fibronectin, extracellular matrix changes, VEGF-induced damage to VSMC vascular permeability, cellular growth, inhibition of protein kinase B (PKB, also known as Akt) activation, angiogenesis, and decrease of Na^+^-K^+^-ATPase activity 79-81. These changes can further lead to endothelial function injury. In addition, fluctuating plasma glucose levels in people with diabetes can also cause endothelial cell damage 82. It is speculated that endoglin is involved in the regulation of ED, and oxidative stress may play a role in this regulation 83. Through suppression of phosphatidylinositol 3 kinase (PI3K)-Akt signalling and activation of FOXO1 in cultured primary endothelial cells, hyperglycemia stimulated placenta growth factor (PlGF) secretion which is a pro-inflammatory angiogenic mediator 84. Hyperglycemia drives ED via different mechanisms, including excessive oxidative stress, over activated inflammation and reduced NO bioavailability (see Figure 2). Mechanistically, metformin improves ED through liver kinase B1 (LKB1)/AMPK and AMPK-independent targets 85. The mechnisms involved can interact with each other and amplify the influence, which may bring a vicious circle.

Previous studies have shown that hyperglycemia-induced oxidative stress could cause deoxyribonucleic acid (DNA) damage and induce endothelial cell senescence, injury, apoptosis, and endothelial-to-mesenchymal transition (EndoMT) 86-88. Upon DNA damage, the nuclear enzyme poly ADP-ribose polymerase (PARP) is activated, promoting the production of adenosine diphosphate (ADP)-ribose polymers. Then, hyperglycemia-induced overproduction of superoxide also inhibits glyceraldehyde phosphate dehydrogenase (GAPDH) activity 89. Furthermore, glycolytic intermediates produce to diacylglycerol, which can activate both classical and novel PKC pathways 81 involved in vascular hyperpermeability, cell growth, neovascularization and other vascular abnormalities of diabetes. There are at least 11 PKC isoforms, and the β- and δ-isoforms appear to be activated preferentially by the diabetic milieu 81. High glucose-induced nuclear factor-kappaB (NF-κB) DNA binding activity may also induce a low-grade pro-inflammatory state and mediate this inhibition of cellular migration by regulating the NO levels in endothelial cells 90, 91. In addition, the release of endothelial cell-derived vasoconstrictor factors and the disruption of the endothelial glycocalyx both increase in a high-glucose environment 92, 93. The main precursor of AGEs, methylglyoxal (MGO), a reactive dicarbonyl generated is believed to play a critical role in ED. As presented in a previous study, after treatment with MGO for three months, it was observed that the formation of AGEs and the expression of monocyte chemoattractant protein 1 (MCP1) and the receptor for AGEs (RAGE) were increased compared to the matched control group 94, while the bioavailability of NO was decreased significantly concurrent with increased level of superoxide anion. Therefore, MGO scavengers have the potential to attenuate hyperglycemia-induced ED and MGO through ATP-sensitive potassium (K_ATP_)/mitogen-activated protein kinase (MAPK) pathway activation 95.

### Insulin resistance (IR) and ED

In diabetes mellitus, hyperinsulinemia and hyperglycemia are both reported to selectively impair receptor-dependent, endothelium-dependent relaxation 96. The metabolic disorders of IR, hyperglycemia and excessive circulating free fatty acids cause vascular tissue oxidative stress, mild inflammation, platelet hyperactivity, and ED. In 1997, Pinkney et al. proposed that the IR syndrome was accompanied by many aspects of atherogenesis, which could be viewed as the different consequences of the effect of ED on different vascular beds 97. In a rat model of IR, the endothelium-dependent relaxation is impaired due to the defect in NO/prostanoid-independent relaxation 98. Associated with lipotoxicity, glucotoxicity, and inflammation, IR may damage the endothelial cell and thus trigger and accelerate atherosclerosis and CVD 30, 99-102.

Insulin activates the PI3K-Akt pathway, phosphorylates eNOS at Serine 1177 site, thereby promoting the synthesis of NO in endothelial cells under normal physiological conditions 103. However, when the body is in a state of IR, the PI3K-Akt pathway is impaired, as well as the process of NO production. Alternatively, the MAPK pathway causes the synthesis of inflammatory markers, including PAI-1, intercellular adhesion molecule 1 (ICAM1), E-selectin, and vascular cell adhesion molecule 1 (VCAM1), thereby leading to ED 104. Therefore, the pathogenic factors of ED induced by obesity, one of the main causes of IR, in the early stages will further impair the insulin signaling pathways of endothelial cells, resulting in reduced vasodilatation, abnormal capillary recruitment, and the transfer of insulin substrate to the target tissues 105. Even after blood glucose levels have returned to normal, the known phenomenon of 'metabolic memory' persists and the harmful effects of hyperglycemia are perpetuated. In addition, molecular and vascular abnormalities involving NO persisted for several months even though the biochemical parameters of IR and ED were normalized, confirming the long-term effects of metabolic memory 106. Increasing evidence shows that chronic inflammation of metabolic tissues plays a causal role in obesity-induced IR. However, it is still unclear how specific endothelial factors affect metabolic tissues 107.

### Advanced glycation end products (AGEs) and ED

The reactive oxygen intermediates (ROI), the reactive chemical entities accelerating the formation of AGEs, activate multiple mechanisms that are potentially involved in the development of vascular complications, such as the activation of NF-κB, PKC and upregulation of the hexosamine pathway 108. The binding of AGEs to RAGE increases intracellular enzymatic superoxide production 109 and induces the expression of heparanase in human umbilical vein endothelial cells (HUVECs), via activation of the forkhead box O4 (FOXO4) 110. In a cohort study, independent of other cardiovascular risk factors, the elevated serum AGEs levels in patients with T2D was shown to be associated with ED 111. AGEs impair endothelial-dependent vascular relaxation and the mechanism is primarily due to reduced bioavailability and activity of NO 112 and activation of p38 and ERK1/2 by reducing eNOS expression and increasing the level of reactive oxygen species (ROS) production 113. Of note, serum AGEs do not follow the same time course even though glycated hemoglobin A1c and markers of endothelial function improve under insulin therapy 114, suggesting that there may be other factors affecting AGEs, in addition to hyperglycemia, such as oxidative stress.

More recently, mounting evidence has supported the contention that microRNAs (miRNAs) and long noncoding RNAs (lncRNAs) are associated with ED 115-118. In this regard, AGEs induce downregulation of miR-200c and miR-200b, which leads to increased expression of target genes Rho-associated coiled-coil kinase (ROCK) and RhoA, respectively 119.

### Oxidative stress, inflammation and ED

Oxidative stress is a state of imbalance between antioxidant systems and exposure to toxic ROS and related chemical species. It has been established that a low concentration of intracellular ROS is essential in maintaining antioxidant/redox homeostasis, vascular integrity and function. However, under pathological conditions such as occuring during the development of atherosclerosis, injury after ischemia-reperfusion and hypoxia, the ROS level is elevated in response to adverse stimuli 120. ROS are produced by a variety of oxidase enzymes, including nicotinamide adenine dinucleotide phosphate (NADPH) oxidase (NOX), lipoxygenase, xanthine oxidase, cyclooxygenase, glucose oxidase, eNOS uncoupling, and mitochondrial electron transport (cytochromeP450 monooxygenase) 121-128. It follows that proteins and lipids are oxidized by excessive ROS (especially free radicals), which leads to the overexpression of redox genes, intracellular calcium overload and DNA fragmentation, and consequently damage to VSMCs, endothelial cells and myocardial cells 129. In the early stage of atherosclerosis, the number of inflammatory cells (monocytes/macrophages/T-lymphocytes) and the process of arteriosclerosis are all associated with enhanced serum levels of inflammatory parameters 130. As seen in chronic inflammation, the arteriosclerotic artery produces different adhesion molecules, cytokines, and growth factors 20, 131. When endothelial cells are activated by inflammation, the increased expression of VCAM1, selectins and ICAM1 will promote monocyte adhesion to the vessel wall 132. Many cytokines, including circulating inflammatory cytokines, tumor necrosis factor-α (TNF-α), ROS, oxidized low-density lipoprotein (oxLDL), and traditional risk factors can directly or indirectly induce abnormal functioning of endothelial cells, resulting in impaired vasodilation, increased endothelial permeability, increased leukocyte adhesion and thrombosis 133-135.

Over-inflammatory/oxidative stress causes ED in patients with and without diabetes 136, 137. Indeed, in young women without IHD and low risk of CVD (“low risk subjects”), the over-inflammatory/oxidative stress may be the main determinant factors of ED and major adverse cardiovascular events (MACE) 137. It is relevant to note that ED could be linked to other non-classical CVDs' risk factors as adipose tissue deposition and excess. This is observed in women 137 with different classes of breast fat density (lowest breast density) and increased risk of pre-diabetes and diabetes. On the other hand, it has been remarked in overweight patients with altered glycemia and IR, as those with pre-DM and without first clinical manifestation of CVD. Excess of abdominal adipose tissue in these overweight pre-diabetics patients with IR is a chronic source of inflammatory molecules that cause ED and reduction of cardiac performance and worse prognosis 138, 139.

In 2002, a study from Ceriello et al. 74 showed that oxidative stress was a common mediator of postprandial hyperglycemia and hypertriglyceridemia on endothelial function. Hyperglycemia increases intracellular ROS through the interrogation mechanism between cytosolic and mitochondrial ROS production 140, which once again shows that the decrease in NO production and the imbalance of increased ROS production may be related to the impaired endothelium-dependent vasodilation in patients with CVD. Conditions such as hyperlipidemia can also induce increased levels of AGEs, leading to increased ROS and oxLDL accumulation in diabetic animals 141. Cytokines and oxLDL stimulate endothelial cell permeability, and NF-κB-dependent inflammatory gene expression and ROS appear to play a central role in mediating both responses 72. Therefore, the vicious cycle among ED, oxidative stress and inflammation will lead to atherosclerosis in diabetes and associated conditions 128.

### Endothelial-to-mesenchymal transition (EndoMT) and ED

EndoMT is an extremely complex pathophysiological process during which endothelial cells lose their specific markers and acquire the phenotypes of mesenchymal cells or myofibroblasts 142. In hyperglycemia, it is deduced that high glucose levels induce EndoMT through ERK, Smad2/3, nucleotide-binding oligomerization domain-containing protein 2 (NOD2), ROCK1, and serum response factor in glomerular endothelial cells - this sequence results in increased expression of mesenchymal markers in different types of endothelial cells 143, 144. Recently, evidence has suggested that EndoMT is a critical link in the interactions between ED and inflammation. Through this process, varieties of inflammatory mediators activate endothelial cells and transform them into mesenchymal-like cells. The factors implicated in this response are interleukin-1beta (IL-1β), TNF-α, NF-κB transcription factor, and endotoxins 145-147. In addition to inflammation and hyperglycemia, hypoxia 148, dyslipidemia 149, miRNAs and lncRNAs 150 and other abnormalities are related to the induction of EndoMT. Notwithstanding these findings, the totalities of the mechanisms involved in EndoMT are not fully understood.

## Current status of metformin in the amelioration of ED

Metformin is a biguanide derivative that exerts an anti-hyperglycemic effect with minimal risk of hypoglycemia. Metformin is currently the first choice for the majority of patients with newly diagnosed T2D requiring medical therapy. The primary status of metformin has arisen from the results of clinical evidence from the UKPDS 34. The reduction of glucose production in liver cells and increased uptake in skeletal muscle are considered the primary processes mediating the hypoglycemic effect of metformin although multiple other actions have been suggested 151.

As for the efficacy of SGLT2i 37 and GLP-1RAs 36, numerous clinical studies have shown that metformin can improve vascular endothelial function and reduce CVD in patients with diabetes (see Table 1 152-167). A study reported in 2001, utilizing a moderate dose of metformin (500 mg twice daily) administered for 12 weeks and compared to placebo, showed improvement in Ach-stimulated flow (and IR) was observed among diet-treated T2D patients. In this study IR was the sole predictor of endothelium-dependent blood flow after metformin treatment 152. Similar benefits were observed with longer-term and higher doses of metformin. Fifteen T2D patients took 1700 mg per day for three months, and t-PA, VCAM1 and ICAM1 levels were significantly reduced 155. For obese patients who have previously received medical nutrition guidance and regular exercise, after taking metformin for 12 weeks (average dosage 1381 ± 85 mg/d), a decrease in PAI-1 and VEGF was observed, effects which were independent of favourable effects of metformin on body mass index (BMI) and glycemic control 156. Among T2D patients with stable coronary heart disease, treatment with metformin affects VCAM1 and asymmetric dimethylarginine (ADMA) levels 166. Still, it does not impair the endothelial healing of drug-eluting stents in these patients with or without insulin use 168. In the long-term treatment (4.3 years) with metformin plus insulin, most of the inflammatory biomarkers, such as the von Willebrand factor (vWF), soluble VCAM1, t-PA, and ICAM1, were significantly reduced even after adjusting for baseline differences in age, sex, smoking and severity of previous CVD 161. When comparing the effect with the other antihyperglycemic agents, at equivalent levels of glycemic control, metformin was more effective in reducing biomarkers reflecting inflammation and improving endothelial functions than repaglinide (an insulin secretagogue) 157, and only rosiglitazone (the insulin-sensitizing agent) improved endothelium-dependent vasodilatation and insulin sensitivity more than metformin 169. In addition, with lipid-lowering agents, such as atorvastatin or fenofibrate, used in combination among T2D patients, metformin is demonstrated not only to prevent the glucose-induced impairment of endothelial functions partly but also potentiates lymphocyte-suppressing, endothelial protective and systemic anti-inflammatory effects of fenofibrate, superior to monotherapy 158, 170. A recent randomized controlled trial showed that through distinct or complementary mechanisms of action on the vascular wall, metformin was able to improve functional capillary density during post-occlusive reactive hyperemia in obese newly diagnosed drug-naive women with T2DM 165.

The traditional paradigms of T2D occurring only in adults and T1D only in children are no longer accurate, as both diseases occur in both age-groups. There is less data available about metformin affects on the endothelium among patients with T1D in whom increasing evidence suggests the deleterious effect of glycemic variability and fluctuations on CVD. The Reversing with Metformin Vascular Adverse Lesions (REMOVAL) trial, the largest and longest trial of metformin in T1D to date, has demonstrated that metformin does not reduce either the carotid intima-media thickness (IMT) or the reactive hyperemia index (RHI) after treatment with metformin for approximately three years 167. However, among the uncomplicated subjects with T1D, metformin is shown to improve the flow mediated dilation (FMD) and the biomarker of oxidative stress (urinary 8-iso-prostaglandin F2α) after 6-month treatment, irrespective of its effects on glycemic control and BMI 160. There was a cardioprotective effect of metformin for those without overt CVD through improving circulating EPCs, pro-angiogenic cells, and circulating endothelial cells count and function independently 171. In addition, with the combination of metformin and the SGLT2i, empagliflozin, used in patients with T1D, arterial stiffness and endothelial function assessed by FMD and RHI were significantly improved in the combination therapy group compared to those on monotherapy 164.

Furthermore, metformin reduces the inflammatory biomarkers, such as vWF, sVCAM1, and sICAM1, among volunteers with impaired glucose tolerance 172. Moreover, metformin may improve vascular function and decrease myocardial ischemia in nondiabetic women with chest pain and angiographically normal coronary arteries 173. Among women with polycystic ovary syndrome (PCOS), those resulting from the critical pathophysiologic component contributing to hyperandrogenisms and the reproductive metabolic features with IR, conflicting results have been reported regarding the effects of metformin on endothelial function 174. Metformin (850-1700 mg daily) therapy could reduce the baseline diameter of the brachial artery, FMD, IMT, and serum VEGFB after reactive hyperemia among women with PCOS, and even improve the FMD values, plasma ET-1 after 6-month treatment 175-179. In one study which compared the characteristics among nonobese adolescents with PCOS and healthy age-matched volunteers, only the surrogate markers of cardiovascular risk such as blood pressure, levels of C-reactive protein (CRP), and PAI-1, whereas the deterioration of vascular structure and function has also been undetected even after 6-month treatment with metformin 180. Metformin therapy reduces the high risk of cardiovascular events in pre-DM patients by reducing coronary ED with two-year therapy 181.

## Pharmacological effects of metformin on endothelial function

### Regulation of vascular tone

Impaired endothelium-dependent vasodilation is a common characteristic in arteries in both T1D and T2D. NO is the major vasodilator synthesized from its precursor L-arginine via eNOS 182. Metformin (60 mg/kg/d) treatment improves endothelial functions in the T2D rat model (Goto-Kakizaki rats) and significantly improves NO bioavailability in rats 183. Metformin (300 mg/kg/d) therapy does not effectively correct hyperglycemia; however, the reduction of vasodilation and total eNOS activity in a T1D rat model (streptozotocin, STZ-induced rats) can be corrected by metformin treatment 184. In a 2006 study, AMPK was found to be necessary for metformin to enhance eNOS activation *in vivo* 35. There is a positive correlation between the concentrations of metformin (50-500 mmol/L) and the levels of eNOS with serine-1179 (Ser1179) phosphorylation and heat shock protein 90 (HSP90), resulting in increased eNOS activation and NO bioactivity 35. Henceforth, AMPK activation became one main recognised mechanisms of the vascular protective effects of metformin. Recently, the eNOS activating effect of metformin (250 mg/kg/d) on EPCs of STZ-induced diabetic mice has also been demonstrated 185.

In diabetes, part of the eNOS process produces superoxide anion instead of NO, and this is referred to as “eNOS uncoupling”. The decrease of endothelium-derived tetrahydrobiopterin (BH4, a vital cofactor for eNOS) promotes the uncoupling of eNOS 50, 51. Metformin (300 mg/kg/d) treatment normalizes Ach-induced endothelial relaxation and increased GTP cyclohydrolase 1 (GCH1, the rate-limiting enzyme in BH4 biosynthesis) and BH4 levels 186 in both diabetic and wild type mice by slowing down the degradation of GCH1 via a post-translational mechanism 187. Metformin (2 mmol/L, 200 mg/kg/d, respectively) may also inhibit NOX (p47-phox) via AMPK activation in HUVECs 188 and increase the phosphorylation of AMPKα and PARP1 in the aortas of spontaneously hypertensive rats 189. Metformin (100-500 mg/kg/d) treatment increases NO bioavailability and attenuates high-sensitivity CRP (hCRP)-induced hypertension by activating of AMPK/peroxisome proliferator-activated receptor δ (PPARδ) pathway 190 and AMPK-eNOS phosphorylation pathway 191 in animal studies. Metformin (500 μM) also reduces p38 MAPK signal transduction through an AMPK dependent mechanism 192.

Metformin affects the expression of several vasoconstrictive molecules such as ET-1, AngII, and tissue factors to regulate vascular tone. In both clinical and basic research, metformin treatment reduces ET-1 levels in patients with PCOS (Diamanti-Kandarakis et al. 2001) and insulin-resistant human endothelial cells 193. Metformin inhibits PKC membrane translocation and activity induced by AngII 194. AngII stimulates vascular NOX and is activated through the AngII receptor AT1R-PKC pathway 195, and is closely related to vascular oxidative stress in ED under diabetic conditions 196. In addition, by inhibiting vasoconstrictor prostanoids, metformin also improves the endothelial function of mesenteric arteries in a T2D rat model 197.

### Inhibition of inflammation and leukocyte adhesion to endothelial cells

**Figure 3:**
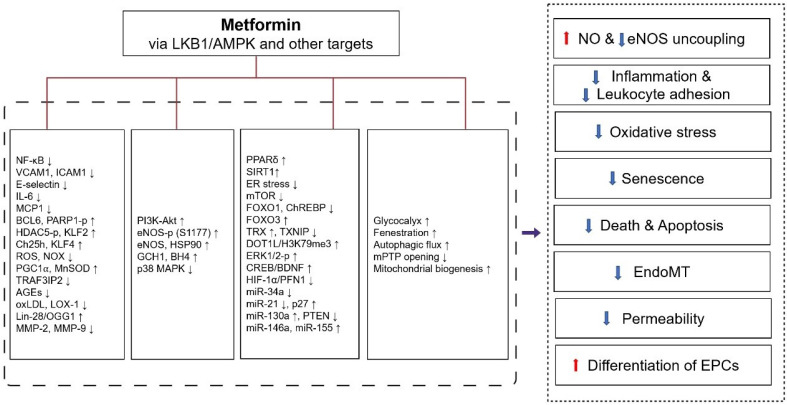
** Protective effects of metformin against endothelial dysfunction and its molecular targets.** Metformin improves endothelial dysfunction through the following mechanisms: 1) increasing NO production and inhibiting eNOS uncoupling, 2) inhibiting inflammation and leukocyte adhesion to endothelial cells, 3) inhibiting oxidative stress, 4) inhibiting endothelial cell senescence, 5) preventing endothelial cell death and apoptosis, 6) inhibition of EndoMT, 7) inhibition of endothelial permeability, 8) increasing differentiation of EPCs. These pharmacological effects of metformin were exerted through LKB1/AMPK and AMPK-independent targets. Abbreviations: AMPK, 5'-adenosine monophosphate-activated protein kinase; AGEs, advanced glycation end products; BCL6, B-cell lymphoma 6; BDNF, brain-derived neurotrophic factor; BH4, tetrahydrobiopterin; ChREBP, carbohydrate response element-binding protein; CREB, cyclic AMP response element binding; Ch25h, cholesterol-25-hydroxylase; DOT1L, Dot1-like protein; ER stress, endoplasmic reticulum stress; ERK, extracellular-signal-regulated kinase; eNOS, endothelial nitric oxide synthase; EPCs, endothelial progenitor cells; EndoMT, endothelial-to-mesenchymal transition; FOXO1, forkhead box O1; FOXO3, forkhead box O3; GCH1, Gtp cyclohydrolase 1; H3K79me3, histone H3 lysine 79 trimethylation; HSP90, heat shock protein 90; HDAC5-p, phosphorylation of histone deacetylase 5; HIF-1α, hypoxia inducible factor-1 alpha; ICAM1, intercellular adhesion molecule 1; IL-6, interleukin-6; LOX-1, lectin-like oxidized LDL receptor 1; KLF2, krüppel-like factor 2; KLF4, krüppel-like factor 4; LKB1, liver kinase B1; mTOR, mammalian target of rapamycin; MnSOD, manganese superoxide dismutase; MMPs, matrix metalloproteinases; mPTP, mitochondrial permeability transition pore; MAPK, mitogen-activated protein kinase; MCP1, monocyte chemoattractant protein 1; MiR, microRNA; NF-κB, nuclear factor-kappaB; NO, nitric oxide; NOX, NADPH oxidase; OGG1, 8-oxoguanine glycosylase 1; oxLDL, oxidized low-density lipoprotein; PPARδ, peroxisome proliferator-activated receptor δ; PGC1⍺, peroxisome proliferator-activated receptor-gamma coactivator 1⍺; PI3K, phosphatidylinositol 3 kinase; PARP1-p, phosphorylation of poly ADP-ribose polymerase 1; PFN1, profilin-1; PTEN, phosphatase and tensin homolog; ROS, reactive oxygen species; SIRT1, sirtuin 1; TRAF3IP2, TRAF3-interacting protein 2; TRX, thioredoxin; TXNIP, TRX-interacting protein; VCAM1, vascular cell adhesion molecule 1.

Metformin inhibits the pro-inflammatory changes induced by cytokines, which may be one mechanism for its vascular actions beyond the hypoglycemic effect. Like the AMPK activator AICAR, which can inhibit the increase in NF-κB reporter gene expression incubated with TNF-α in HUVECs 198, metformin (2-10 mM) is been found to inhibit inflammation through the AMPK-dependent IKK/IKBα/ NF-κB inhibitory pathway (Figure 3) 199. Except for inhibition of NF-κB through blockade of the PI3K-Akt pathway in human saphenous vein endothelial cells 200, metformin also inhibits TNF-α induced production of Interleukin-6 (IL-6) 201 and several other inflammatory molecules responsible for monocyte adhesion to activated endothelial cells including VCAM1, MCP1, E-selectin, and ICAM1, by up-regulating B-cell lymphoma 6 (BCL6) and AMPK-induced phosphorylation of PARP1 at ser-177 202, and the phosphorylation of histone deacetylase 5 (HDAC5) at serine 498 203.

Treatment with metformin decreases retinal leukocyte adhesion 204, palmitic acid-induced monocyte adhesion 205, and reduces clinically relevant high levels of CRP-induced ED and hypertension 191. Metformin (300-500 mg/kg/d) pretreatment also reduces ferrous chloride-induced thrombosis in carotid arteries 206. Metformin can also reduces the uptake of oxLDL by endothelial cells and reduces subsequent inflammatory signals, thereby preventing macrophage adhesion and infiltration 207. Additional mechanisms of metformin-stimulated autophagic flux 205 and krüppel-like factor 2 (KLF2) restoration 203, 208 partially contribute to the anti-inflammatory action of metformin in endothelial cells.

### Inhibition of oxidative stress in endothelial cells

Metformin inhibits ROS production and reduces ROS levels in endothelial cells through multiple antioxidant mechanisms, including inhibiting NOX and stimulating catalase activity 209 and inhibiting lectin-like oxidized LDL receptor 1 (LOX-1) expression 210. Other antioxidant mechanisms include reducing PKC membrane translocation and activity 194, increasing peroxisome proliferator-activated receptor-gamma coactivator 1α (PGC1α) 211 and thioredoxin (TRX) expression 212, and normalizing mitochondrial ROS production 211. Some evidence suggests that PGC1α is an essential regulator of intracellular ROS levels. Metformin can induce PGC1α and manganese superoxide dismutase (MnSOD), resulting in inhibition of mitochondrial ROS 213 and increased TRX expression through activation of the AMPK-forkhead box O3 (FOXO3) pathway in human aortic endothelial cells (HAECs) 212. In addition, metformin therapy inhibits endoplasmic reticulum stress (ER stress) and oxidative stress on activation of the AMPK/PPARδ pathway in obese diabetic mice 190. ER stress plays a key role in progression of diabetes and development of complications, especially CVD 214. While oxLDL is a product of chronic oxidative stress which generate pro-oxidant effects by inducing ROS generation, metformin (20 μM) decreases ROS levels by inhibiting LOX-1 expression and increasing Akt/eNOS levels 210.

Besides its effect on oxidative stress, metformin also reduces nitroxidative stress. The treatment of metformin (300 mg/kg/d) is observed to increase the production of NO by 37% and 57% in aortic and glomerular endothelial cells of experimental rats, respectively, while decreasing ONOO (-) (cytotoxic peroxynitrite) by 32% and 34%, compared with controlled animals 215. Moreover, as the main DNA glycosylase, 8-oxoguanine glycosylase 1 (OGG1) can resist ROS and is involved in various vascular diseases. The level of OGG1 increases under metformin (0.5 mM) treatment through the AMPK/Lin-28/OGG1 pathway 216. The anti-oxidative function of metformin (10 μM-1 mM) also relates to inhibition of TRAF3-interacting protein 2 (TRAF3IP2), which is a redox-sensitive cytoplasmic adapter protein functions upstream of IKK/NF-κB and c-Jun N-terminal kinase (JNK)/AP-1 (Activator protein-1) 217.

### Inhibition of endothelial cell senescence

Endothelial cell senescence leads to ED and contributes to the progression of age-associated vascular disorders 218. Endothelial cell senescence is characterized by cell cycle arrest and pro-inflammatory gene expression 219-221 and is driven by many factors, including high glucose, ROS, inflammatory cytokines, ionizing radiation, and telomere dysfunction 222, 223. High glucose induces endothelial cell senescence by inhibiting sirtuin 1 (SIRT1) expression, which can be attenuated with metformin (50-250 μM) treatment by modulating the SIRT1 downstream targets forkhead box O1 (FOXO1) and p53/p21 224, 225. In ApoE^-/-^ mice, metformin (50 mg/kg/d) therapy significantly reduces vascular aging and inhibits atherosclerotic plaque formation through AMPK activation leading to SIRT1/Dot1-like protein (DOT1L)/ histone H3 lysine 79 trimethylation (H3K79me3)-induced upregulation of sirtuin 3 (SIRT3) levels 226. Metformin increases fenestrations in liver sinusoidal endothelial cells isolated form old and young mice 227. Calcification is a vascular disease marker and a prognostic factor of CVD, which is related to the aging of the vascular system 228. It is speculated that metformin, acting via AMPK dependent mechanisms, may be a potential target for the treatment of vascular calcification 229. Indeed, clinical trials have been designed to assess the potential benefits of metformin as an anti-aging drug that enhances healthspan and extends lifespan 230. The beneficial effects of metformin on aging and healthspan are primarily indirect via its effects on cellular metabolism and result from its anti-hyperglycemic action, enhancing insulin sensitivity, reduction of oxidative stress and protective effects on the endothelium and vascular function 230.

### Inhibition of endothelial cell death and apoptosis

Metformin (100 μM) prevents endothelial cell death by inhibiting mitochondrial permeability transition pore (mPTP), which is a mitochondrial channel involved in cell death 231. Metformin partly protects endothelial cells from dysfunction and apoptosis via reducing p38 MAPK phosphorylation 192, inhibiting the mammalian target of rapamycin (mTOR) pathway 232, promoting the PI3K-Akt and phospho-ERK 1/2 pathways 233, and down regulation of TRX-interacting protein (TXNIP) transcription by inactivating carbohydrate response element-binding protein (ChREBP) and FOXO1 234 in endothelial cells. Metformin (0.01-1.0 mmol/L) also inhibits endothelial cell apoptosis via upregulation of VEGF receptors, fatty acid binding protein 4 (FABP4) 235 and the AMPK/ cyclic AMP response element binding (CREB)/brain-derived neurotrophic factor (BDNF) pathway 236. Metformin (100 μmol/L or 10 mmol/L) can also inhibit mPTP (a sensitive channel) opening in three endothelial cell types, including HMEC-1, HUVECs, and bovine aortic endothelial cells (BAECs), from preventing hyperglycemia-induced cell death 231.

### Inhibition of EndoMT

EndoMT is a driver event in the pathogenesis of atherosclerosis, diabetes associated myocardial remodeling, pulmonary hypertension and others. A study in 2018 explored the effects of different flow patterns on the EndoMT in endothelial cells, indicating that pharmacological activation of AMPK or SIRT1 could attenuate oscillatory shear stress (OS)-induced EndoMT 237. The beneficial therapeutic effect of metformin on atherosclerosis may be mediated at least in part by inhibiting the EndoMT in the vasculature. The expression of Krüppel-like factor 4 (KLF4), a major transcription factor showing endothelial protection and maintenance of vascular homeostasis, and cholesterol-25-hydroxylase (Ch25h) decreases in high glucose level 238, while metformin (200 mg/kg/d) can increase the expression of KLF4 and Ch25h to suppress EndoMT 239.

### Inhibition of the permeability of the endothelium

The increased permeability of the endothelium is accompanied by inflammation-mediated reversible cell rounding and inter-endothelial gap formation 240 and high glucose-activated PKC pathway 241. Metformin (0.33 mg/ml, two weeks) treatment is associated with improved glycocalyx barrier properties and indicates reduced vascular permeability in mice 242. Metformin (400 mg/kg/d, 100 mg/kg/d, respectively) administration is associated with reduced lung endothelial hyperpermeability and systemic inflammatory response in STZ-induced diabetic mice 243 and alleviates microvascular ED by suppressing hypoxia inducible factor-1 alpha (HIF-1α)/Profilin-1 (PFN1) signaling, which mediates endothelial cell permeability during diabetic retinopathy 244. Moderate doses of metformin (0.1-1.0 mM) are shown to produce an increase in the trans-endothelial resistance of endothelial monolayers and enhance the vascular barrier integrity 245. By activation of AMPK signaling pathway and regulation of oxidant/anti-oxidant balance, metformin abrogates the disruptive effect of polyphosphate which induce hyper-permeability and inflammatory responses through mTOR pathway in endothelial cells 246.

### Increased differentiation of endothelial progenitor cells (EPCs)

By restoring damaged endothelial cells, EPCs represent new targets for treating vascular diseases 247, 248. EPCs, originating from bone marrow 249 and circulating in peripheral blood, have the ability of endothelial differentiation and secretion of angiogenic growth factors and cytokines 250. Metformin treatment significantly increases EPC differentiation *in vitro* (1 mM) 251 and in diabetic mice (250 mg/kg/d) 185. As to mechanism, increased levels of AMPK and eNOS phosphorylation 185, light chain3 (LC3) expression and NO production, and decreased mTOR, p70 S6K, TGF-β expression 251 and MMP-2 and MMP-9 expression 252 are found in EPCs. In addition, the effects of metformin on improving EPC dysfunction are mediated by several miRNAs, such as miR‑130a/ phosphatase and tensin homolog (PTEN) 253 and miR-221/p27 254.

## Future directions

### Epigenetic effects of metformin

MicroRNAs (miRNAs) are highly conserved, small, and noncoding RNAs involved in the post-transcriptional regulation of gene expression. MiRNAs participate in regulating endothelial function and dysfunction. Diabetes and hyperlipidemia-induced inflammatory responses upregulate the expression of connexins and Rho kinase by selective downregulation of miR-10a, miR-139b, miR-206, and miR-222 116. A study in 2006 found that metformin (50 μM), or the inhibition of miR-34a by treatment with an anti-miR-34a inhibitor, increased the expression of SIRT1 and attenuated the impairment in angiogenesis in high glucose-exposed mouse microvascular endothelial cells (MMECs) 255. In metformin (0-10 mM) or miR-221 siRNA-treated EPCs, AMPK inhibition decreased the expression of p27 and AMPK-mediated autophagic activity 254. In contrast, upregulation of miR‑130a 253, miR-146a and miR-155 256 via the treatment of metformin (50 μM, 200 mg/kg/d) is observed in PA‑exposed EPCs and endothelium, respectively. Giuliani et al. 257 investigated the miRNA landscape in endothelial cells subject to replicative senescence after a long-term application with metformin (20 μM), and found the differential expression of 27 miRNAs (including miR-100-5p, -125b-5p, -654-3p, -217 and -216a-3p/5p).

Long noncoding RNAs (lncRNAs) represent a family of non-protein-coding transcripts (>200 nucleotides), which occupy the majority of the human genome 258, while circular RNAs (circRNAs) are a large category of noncoding RNAs widely expressed in eukaryotic cells 259. Growing evidence suggests that lncRNAs 260-263 and circular RNAs 264-268 regulate endothelial functions, while the role of lncRNAs and circular RNAs in the protective effect of metformin on endothelial cells remains largely unclear.

### Metformin on endothelial cell metabolism

Evidence has demonstrated the importance of endothelial cells in the maintenance of homeostasis across the entire vascular system. The initiation and progression of atherosclerosis may originate from the disorder of endothelium intracellular metabolism that can be detected at the earliest stages of developing the syndrome 269. Endothelial cell metabolism is important for maintaining metabolic health, such as glucose, lipid and amino acid, as they have been most extensively studied 270. The study identifies a metabolic program that promotes the acquisition of a quiescent endothelial state and emphasizes the role of metabolites as signaling molecules in endothelial cells 271. The activity and migration of endothelial cells decrease when the enzymes related to glucose metabolisms, such as G6P dehydrogenase and transketolase, are inhibited 272. Previous studies showed that metformin affects FABP4 mediated endothelial fatty acid metabolism 273, 274. Endothelial cells internalize chylomicrons, which trigger lipid accumulation in aortic of lipoprotein lipase-deficient mice 275. Recently, metformin (100 μM) is shown to reduce saturated fatty acid-induced lipid accumulation and inflammatory response by restoring autophagic flux in endothelial cells 205. In addition, metformin (1-5 mM) takes part in regulation of hexose transport 276, reversing glucose starvation-induced ER stress 277 in endothelial cells. Metformin, GLP-1RAs, and SGLT2i are the only drugs screened in seven classes of anti-hyperglycemic drugs which reduce ER stress caused by pharmacological or hyperglycemic conditions in human coronary artery endothelial cells (HCAECs) 214. In endothelial cells arginine, glutamine, leucine and valine play a role in the metabolism of endothelial cells 278-281.

### Metformin on mitochondrial dynamics

Mitochondria are critical integrators that participate in signal transduction, energy production, ROS generation, and cell apoptosis. Recent studies have highlighted the importance of mitochondrial dynamics, especially mitochondrial fusion and fission in mitochondrial homeostasis 282. In endothelial cells, more fragmented structures caused by fission are associated with mitochondrial dysfunction, contributing to ED with an unclear mechanism 283. Mitochondrial division is triggered when Dynamin-related protein 1 (DRP1), a cytosolic guanosine-5'-triphosphatase, binds with fission 1 (FIS1) or mitochondrial fission factor (MFF) on mitochondria 282. Metformin (300 mg/kg/d) is observed to suppress the progression of atherosclerosis through the inhibition of DRP1-mediated mitochondrial fission in STZ-induced diabetic ApoE^-/-^ mice 284. In addition to inhibiting mitochondrial division, promoting mitochondrial fusion may also benefit endothelial cell function and represent a potential therapeutic approach for diabetes patients with CVD, which is an area that needs further study. In terms of the efficacy of metformin, studies have also shown that metformin (0.01-2 mmol/L) can promote mitochondrial biogenesis by activating AMPK and inducing PGC1α in HUVECs 213.

### Therapeutic potential of metformin in COVID-19 patients with diabetes

Coronavirus disease 19 (COVID-19), caused by the severe acute respiratory syndrome-coronavirus 2 (SARS-CoV-2), is associated with multiple factors, such as cytokine storm, hyperactive inflammatory and immune responses, and coagulation disorders 285. Among COVID-19 patients with diabetes, the intensive care hospitalization rate and mortality rate are two to three times higher than that of patients without diabetes 286. Diabetes has been identified as an independent risk factor for poor prognosis of COVID-19 287, 288. The underlying pathogenic links between COVID-19 and diabetes include effects on inflammation, glucose homeostasis, immune status changes and renin angiotensin aldosterone system (RAAS) activation 289. Metformin treatment is associated with reduced mortality in COVID-19 patients with diabetes 290-292. Patients who used metformin before hospitalization also showed lower severity parameters at admission 293. During SARS-CoV-2 infection, in addition to controlling the glucose level, metformin may attenuate ED, inhibit viral entry and infection, resist oxidative stress, and change inflammatory and immune responses that would explain its ability to achieve cardiovascular protection in COVID-19 294, 295. Therefore, metformin may represent a drug candidate for treating COVID-19 and gain the advantage of fighting SARS-CoV-2 induced immune storm in COVID-19 patients with diabetes 296.

### Metformin on other transcriptional factors

Recent data has shown that islet ED in db/db mice was evident compared with non-diabetic mice 297. Metformin/empagliflozin treatment (6 weeks) could reduce the expression of dysfunction marker genes and increase insulin release *in vivo* 297. It is speculated that metformin may improve the blood glucose level of T2D patients by enhancing the function of islet endothelial cells. Metformin (100 mg/kg/d) can also promote the development of pulmonary vessels and up regulate the expression of glioma-associated oncogene homolog 1 (Gli1) in pulmonary vascular endothelial cells of hyperoxia newborn mice 298.

## Conclusion

Similar as SGLT2i and GLP-1RAs, numerous clinical studies have shown that metformin can improve vascular endothelial functions and reduce CVD in patients with diabetes. Mechanistically, metformin improves ED through LKB1/AMPK and AMPK-independent targets. In addition to several common mechanisms, including increasing NO production, inhibiting inflammation and oxidative stress, metformin can protect endothelial cell functions and the intact endothelium by reducing EndoMT and increasing the differentiation ability of EPCs. Based on the protective effects of metformin on blood vessels summarized in these basic and clinical studies, we suggest that metformin is also worthy of attention for potential treatments for CVD and other panvascular diseases in which ED plays a fundamental role.

## Figures and Tables

**Table 1:** Clinical evidence demonstrating the effect of metformin on endothelial function among diabetic patients

Articles	Subjects	Intervention in metformin	Assessments	Conclusions
Mather, K. J (2001) 152	Diet-treated T2D	1000 mg/d, 12 weeks, N = 29	ACh-stimulated flows:↑; Nitroprusside-stimulated flows: NS; Verapamil-stimulated flows: NS	IR was the sole predictor of endothelium-dependent blood flow following metformin treatment.
Abbasi F (2004) 153	T2D	1000-2000 mg/d, 12 weeks, N = 16	sICAM-1↑; sVCAM-1: NS; ET-1: NS	Metformin, either as monotherapy or in combination with a sulfonylurea drug, led to a decrease in several CVD risk factors in patients with T2D.
De Jager J (2005) 154	T2D treated with insulin	850 mg/d, 16 weeks, N = 196	vWf↓; sET-1↓; t-PA↓; PAI-1↓; s-ICAM-1: NS; s-VCAM-1↓	An improvement of endothelial function with metformin in T2D treated with insulin, which was largely unrelated to changes in glycemic control.
Skrha J (2007) 155	T2D	1700 mg/d, 12 weeks, N = 15	tPA↓; sVCAM-1↓; sICAM-1↓; Microcirculation by laser Doppler: NS	Metformin treatment promotes endothelium effects associated with a complex of metabolic changes in T2D.
Ersoy C (2008) 156	Obese T2D	1381 ± 85 mg/d, 12 weeks, N = 24	PAI-1↑; VEGF↑	A beneficial effect on VEGF and PAI-1 levels with metformin in obese T2D.
Lund SS (2008) 157	Non-obese T2D without insulin	2000 mg/d, 16 weeks, N = 83	PAI-1↓; tPA↓; s ICAM-1↓; sVCAM-1↓; ET-1↓	Metformin was more effective in reducing selected biomarkers reflecting inflammation and endothelial dysfunction compared with repaglinide despite similar glycemic levels between treatments.
Tousoulis D (2010) 158	Newly diagnosed DM	850 mg/d, 6 weeks, N = 15	Resting FBF: NS; EDD: decrease in combination with atorvastatin	Combined with metformin and atorvastatin for 6 weeks partly prevented the glucose-induced impairment of EDD.
Fidan E (2011) 159	T2D	850-2550 mg/d, 12 weeks, N = 20	PAI-1: NS; sICAM-1↓; ET-1: NS; Fibrinogen: NS	Metformin was effective in controlling inflammatory markers in addition to metabolic parameters.
Pitocco D (2013) 160	uncomplicated T1D	2550 mg/d, 6 months, N = 21	FMD↑; NMD: NS	Metformin improved FMD and increased PGF2α in uncomplicated T1D.
de Jager J (2014) 161	T2D treated with insulin	850 mg/d, 4.3 years, N = 131	vWf↓; sVCAM-1↓; s-ICAM-1↓; t-PA, PAI-1↓; ET-1: NS	Metformin is associated with improvement in some markers of endothelial function in T2D.
Kruszelnicka O (2015) 166	T2D with stable CHD	Previous 1 year, N = 40	sVCAM-1 ↓, ADMA ↑	Metformin affects VCAM1 and ADMA levels among T2D patients with stable CHD.
Shigiyama F (2017) 162	T2D treated with metformin	750-1500 mg/d, 16 weeks, N = 54	FMD: NS in alone; increase in combination with linagliptin	Among T2D patients with moderate hyperglycemia, metformin plus linagliptin induced both better glycemic control and improvement of endothelial function.
Kitao N (2017) 163	T2D treated with metformin	1000-1500 mg/d, 12weeks, N = 48	FMD: NS	Combination of vildagliptin and metformin did not affect endothelial function but exert favorable effects on adipokine with T2D without advanced atherosclerosis.
Petrie JR (2017) 167	T1D at increased risk for CVD	2000 mg/d, 3 years, N = 219	Progression of mean cIMT: NS Reactive hyperaemia index: NS	Metformin did not affect on endothelial function but might have a wider role in cardiovascular risk management.
Lunder, M (2018) 164	T1D	2000 mg/d, 12 weeks, N = 10	Beta stiffness: NS in metformin alone; FMD↑	Empagliflozin on top of metformin treatment significantly improved arterial stiffness compared to metformin in T1D.
Schiapaccassa, A (2019) 165	Obesity T2DM women	1700 mg/d, 30 days, N = 19	Nutritive microvascular reactivity↑; Functional capillary density during post-occlusive reactive hyperemia↑	Metformin was able to improve vascular function in obese newly diagnosed drug-naive T2DM women, through distinct or complementary mechanisms of action on the vascular wall.

Abbreviations: Ach, acetylcholine; ADMA, asymmetric dimethylarginine; CHD, coronary heart disease; CVD, cardiovascular Disease; DM, diabetes mellitus; EDD, endothelium-dependent vasodilation; ET-1, endothelin-1; FBF, forearm blood flow; FMD, flow mediated dilation; IR, insulin resistance; NMD, nitroglycerin-mediated dilation; NS, non-significant; PAI-1, plasminogen activator inhibitor-1; PGF2α, prostaglandin F2α; s-ICAM-1, soluble intercellular adhesion molecule-1; s-VCAM-1, soluble vascular cell adhesion molecule-1; T1D, type 1 diabetes; T2D, type 2 diabetes; t-PA, tissue plasminogen activator; VEGF, vascular endothelial growth factor; *v*Wf, von willebrand factor.

## Abbreviations

Ach: Acetylcholine
ADP: Adenosine Diphosphate
AMPK: 5'-Adenosine Monophosphate-activated Protein Kinase
AP-1: Activator Protein-1
ATP: Adenosine Triphosphate
AGEs: Advanced Glycation End Products
AngⅡ: AngiotensinⅡ
ADMA: Asymmetric Dimethylarginine
BCL6: B-Cell Lymphoma 6
BDNF: Brain-Derived Neurotrophic Factor
BH4: Tetrahydrobiopterin
BMI: Body Mass Index
BAECs: Bovine Aortic Endothelial Cells
CGRP: Calcitonin Gene-related Peptide
ChREBP: Carbohydrate Response Element-binding Protein
CO: Carbon Monoxide
CREB: Cyclic AMP Response Element Binding
CVD: Cardiovascular Disease
Ch25h: Cholesterol-25-Hydroxylase
CRP: C-Reactive Protein
DNA: Deoxyribonucleic Acid
DOT1L: Dot1-like Protein
ED: Endothelial Dysfunction
ER stress: Endoplasmic Reticulum Stress
ERK: Extracellular-signal-regulated Kinase
eNOS: Endothelial Nitric Oxide Synthase
EPCs: Endothelial Progenitor Cells
EndoMT: Endothelial-To-Mesenchymal Transition
ET-1: Endothelin 1
FABP4: Fatty Acid Binding Protein 4
FBF: Forearm Blood Flow
FMD: Flow Mediated Dilation
FOXO1: Forkhead Box O1
FOXO3: Forkhead Box O3
FOXO4: Forkhead Box O4
GAPDH: Glyceraldehyde Phosphate Dehydrogenase
Gli1: Glioma-associated Oncogene Homolog 1
GCH1: Gtp Cyclohydrolase 1
GLP-1RAs: Glucagon-like Peptide-1 Receptor Agonists
H3K79me3: Histone H3 Lysine 79 Trimethylation
HSP90: Heat Shock Protein 90
hCRP: High-Sensitivity CRP
HDAC5: Histone Deacetylase 5
HAECs: Human Aortic Endothelial Cells
HCAECs: Human Coronary Artery Endothelial Cells
HIF-1α: Hypoxia Inducible Factor-1 Alpha
HUVECs: Human Umbilical Vein Endothelial Cells
H2S: Hydrogen Sulfide
IR: Insulin Resistance
ICAM1: Intercellular Adhesion Molecule 1
IHD: Ischemic Heart Disease
IL-1β: Interleukin-1Beta
IL-6: Interleukin-6
IMT: Intima-Media Thickness
JNK: C-Jun N-terminal Kinase
KLF2: Krüppel-Like Factor 2
KLF4: Krüppel-Like Factor 4
LC3: Light Chain3
LKB1: Liver Kinase B1
LncRNAs: Long Noncoding RNAs
LOX-1: Lectin-like Oxidized LDL Receptor 1
MGO: Methylglyoxal
mTOR: Mammalian Target Of Rapamycin
MnSOD: Manganese Superoxide Dismutase
MMPs: Matrix Metalloproteinases
mPTP: Mitochondrial Permeability Transition Pore
MAPK: Mitogen-Activated Protein Kinase
MCP1: Monocyte Chemoattractant Protein 1
MiRNAs: MicroRNAs
MMECs: Mouse Microvascular Endothelial Cells
NADPH: Nicotinamide Adenine Dinucleotide Phosphate
NE: Norepinephrine
NF-κB: Nuclear Factor-KappaB
NMD: Nitroglycerin-Mediated Dilation
NO: Nitric Oxide
NOD2: Nucleotide-Binding Oligomerization Domain-Containing Protein 2
NOX: NADPH Oxidase
NPY: Neuropeptide Y
OGG1: 8-Oxoguanine Glycosylase 1
OS: Oscillatory Shear Stress
oxLDL: Oxidized Low-Density Lipoprotein
PFN1: Profilin-1
PPARδ: Peroxisome Proliferator-Activated Receptor δ
PGC1⍺: Peroxisome Proliferator-Activated Receptor-Gamma Coactivator 1α
PI3K: Phosphatidylinositol 3 Kinase
PA: Plasminogen Activator
PAI: Plasminogen Activator Inhibitor
PARP: Poly ADP-ribose Polymerase
PCOS: Polycystic Ovary Syndrome
PlGF: Placenta Growth Factor
PTEN: Phosphatase And Tensin Homolog
PKB, also known as Akt: Protein Kinase B
PKC: Protein Kinase C
RHI: Reactive Hyperemia Index
ROI: Reactive Oxygen Intermediates
ROS: Reactive Oxygen Species
RAGE: Receptor For AGEs
ROCK: Rho-Associated Coiled-Coil Kinase
SGLT2i: Sodium Glucose Cotransporter 2 inhibitors
SIRT1: Sirtuin 1
SIRT3: Sirtuin 3
STEMI: ST-segment Elevation Myocardial Infarction
STZ: Streptozotocin
TRAF3IP2: TRAF3-Interacting Protein 2
TRX: Thioredoxin
TXA2: Thromboxane A2
TIMPs: Tissue Inhibitors Of Metalloproteinases
t-PA: Tissue-PA
TGF-β: Transforming Growth Factor-Β
TXNIP: TRX-Interacting Protein
TNF-α: Tumor Necrosis Factor-Α
T1D: Type 1 Diabetes
T2D: Type 2 Diabetes
UKPDS: UK Prospective Diabetes Study
u-PA: Urokinase-PA
VCAM1: Vascular Cell Adhesion Molecule 1
VEGF: Vascular Endothelial Growth Factor
VSMCs: Vascular Smooth Muscle Cells
vWF: Von Willebrand Factor
